# Crude Astragalus polysaccharides ameliorate cognitive impairment by preserving blood-brain barrier integrity and suppressing GSDMD-mediated pyroptosis in jellyfish-envenomed mice

**DOI:** 10.3389/fphar.2026.1853198

**Published:** 2026-06-08

**Authors:** Zichong Yao, Ruxue Li, Ming Li, Jingwen Liang, Hua Chen, Rui Wang

**Affiliations:** College of Pharmaceutical Sciences & Key Laboratory of Ministry of Education in Protection and Utilization of Medicinal Resources of Liupanshan Area, Ningxia Medical University, Yinchuan, Ningxia, China

**Keywords:** blood-brain barrier, crude Astragalus polysaccharides, jellyfish envenomation, neuroinflammation, pyroptosis

## Abstract

**Introduction:**

Jellyfish envenomation is an escalating global health threat that can induce severe neurotoxic consequences, but effective therapeutic strategies remain limited. This study investigated whether crude astragalus polysaccharides (APS) protect against jellyfish venom-induced cognitive impairment and explored the underlying mechanisms involving blood-brain barrier (BBB) integrity and pyroptosis.

**Methods:**

A murine jellyfish envenomation model was established by intravenous venom injection. Mice were treated with APS, edaravone, or the Caspase-1-specific inhibitor VX-765. Behavioral performance was evaluated using the open field test and Morris water maze. Histopathological injury, cerebral edema, BBB permeability, tight junction protein expression, matrix metalloproteinase-9 (MMP9) expression, inflammasome/pyroptosis-related markers, and inflammatory cytokines were assessed using H&E staining, wet/dry weight measurement, Evans blue extravasation, western blotting, RT-qPCR, and ELISA.

**Results:**

APS ameliorated venom-induced anxiety-like behaviors, locomotor deficits, and spatial memory impairment. Histopathologically, APS preserved hippocampal neuronal integrity and attenuated cerebral edema and hemorrhage. APS also reduced BBB disruption by suppressing MMP9 upregulation and restoring tight junction proteins, including ZO-1, Occludin, and Claudin-5. In addition, APS decreased JNK1 expression and inhibited activation of the NLRP3/Caspase-1/GSDMD pyroptotic pathway, as shown by reduced NLRP3 expression, Caspase-1 cleavage, GSDMD-N formation, and inflammatory cytokine release. VX-765 recapitulated the inhibitory effects of APS on pyroptotic markers and cytokines, including IL-1β, IL-18, and TNF-α. Co-administration of APS and VX-765 produced no additive benefit.

**Discussion:**

These findings indicate that APS protects against jellyfish venom-induced neurotoxicity mainly by preserving BBB integrity and suppressing canonical Caspase-1-dependent GSDMD-mediated pyroptosis. APS may therefore represent a potential therapeutic candidate for marine envenomation-associated neurological injury.

## 1 Introduction

Jellyfish envenomation has emerged as a significant and escalating global public health threat, with a surge in reported cases over the past 2 decades due to climate change, ocean warming, and increased marine recreational activities (Dobson et al., 2024). The severity of jellyfish stings varies considerably across species and geographical regions, with potentially fatal outcomes particularly associated with highly venomous species such as *Chironex fleckeri* and *Carukia barnesi* in Australian waters, *Nemopilema nomurai* in Southeast Asian seas, and *Cyanea capillata* along Chinese coasts (Thaikruea, 2023). The complex composition of jellyfish venom, comprising phospholipase A2 (PLA2), metalloproteinases, pore-forming toxins, and various bioactive peptides (Hwang et al., 2025), contributes to diverse pathophysiological manifestations ranging from local dermatological reactions to severe systemic complications. Among these, the consequences of neurological complications may be overlooked; however, these complications represent the devastating outcomes, as jellyfish venom-induced neurotoxicity can lead to abnormal neuronal excitability, synaptic transmission disruption, neuroinflammation, and progressive neurological deterioration (Yanagihara et al., 2024), significantly impacting patient prognosis and quality of life. The neurotoxic effects involve interference with voltage-gated ion channels, activation of inflammatory cascades, and disruption of cellular homeostasis, which may collectively result in irreversible neuronal damage that current therapeutic interventions fail to adequately address (Li Y. et al., 2025).

Recent advances have been made in understanding the neurotoxic mechanisms of jellyfish venom, primarily focusing on ion channel dysregulation and mitochondrial dysfunction. However, specific mechanisms underlying blood-brain barrier (BBB) damage, cognitive impairment, and potential protective interventions remain insufficiently elucidated. As one of the major components of jellyfish venom, matrix metalloproteinase-9 (MMP9) may play a pivotal role in neuroinflammation propagation and BBB disruption following jellyfish envenomation (Asirvatham et al., 2023). The BBB, serving as a critical protective interface between systemic circulation and the central nervous system, becomes compromised following jellyfish envenomation through MMP9-mediated degradation of tight junction (TJ) proteins such as Occludin, Claudin-5, and ZO-1, yet the specific pathways governing this barrier dysfunction and its relationship to neurological outcomes are poorly understood. MMP9 activation leads to basement membrane component degradation, thus facilitating venom penetration into neural tissues and may trigger inflammatory cascades through NF-κB and MAPK signaling pathways (Zhang et al., 2024). Recent evidence has highlighted the critical role of NLRP3 inflammasome activation in mediating neuroinflammation and pyroptotic cell death, in which the MAPK pathway serves as an upstream regulator of NLRP3 activation (Cai et al., 2022). In response to these pathological mechanisms, Crude astragalus polysaccharides (APS), bioactive compounds extracted from the traditional Chinese medicinal herb Astragalus membranaceus (Huangqi), have been extensively reported to possess potent neuroprotective properties (Sang et al., 2025). Multiple studies have demonstrated that APS can effectively attenuate oxidative stress by scavenging reactive oxygen species and enhancing antioxidant enzyme activities (Xiao et al., 2024), while simultaneously suppressing neuroinflammation through inhibition of microglial activation, NLRP3 inflammasome activation, and inflammatory mediator production, including IL-1β and IL-18 (Wang et al., 2022). These multifaceted pharmacological activities suggest that APS may offer therapeutic benefits in ameliorating jellyfish venom-induced neurotoxicity. Therefore, we hypothesize that APS may alleviate jellyfish venom-induced neurotoxicity and cognitive dysfunction through inhibition of neuroinflammation and pyroptosis.

In this study, intravenous injection of jellyfish venom into mice induced severe anxiety-like behaviors, locomotor deficits, and spatial memory impairment, accompanied by hippocampal neuronal damage, cerebral edema, hemorrhage, and BBB disruption. These pathological changes were substantially ameliorated by APS treatment, with histopathological analysis revealing preserved neuronal architecture and reduced tissue damage. Mechanistically, APS suppressed MMP9 upregulation and restored tight junction protein expression, including ZO-1, Occludin, and Claudin-5, indicating preservation of BBB integrity. APS also reduced JNK1 expression and inhibited the NLRP3/Caspase-1/GSDMD pyroptotic cascade, as evidenced by decreased Caspase-1 cleavage, reduced GSDMD-N formation, and lower IL-1β and IL-18 production. To establish the causal role of Caspase-1-dependent pyroptosis, we employed the Caspase-1-specific inhibitor VX-765. The comparable effects of APS and VX-765, together with the absence of additive benefit upon co-administration, indicated that APS exerts its neuroprotective effects primarily through inhibition of the Caspase-1-dependent pyroptotic pathway. Collectively, these findings suggest that the neuroprotective effects of APS against jellyfish venom-induced neurotoxicity are mediated through dual mechanisms involving BBB preservation and suppression of GSDMD-mediated pyroptosis. Therefore, targeting BBB disruption and Caspase-1/GSDMD-mediated pyroptosis may represent a promising therapeutic strategy for alleviating neurological complications associated with marine envenomation.

## 2 Materials and methods

### 2.1 Reagents

Antibodies against MMP9, ZO-1, Occludin, Claudin-5, NLRP3, GSDMD-N, IL-1β, IL-18, IL-6, JNK1, β-tubulin, and GAPDH were purchased from Affinity Biosciences (Jiangsu, China). Mouse IL-1β, IL-18, and TNF-α ELISA kits were purchased from Boshen Biotechnology Co., Ltd. (Jiangsu, China). The Caspase-1 inhibitor VX-765 (Belnacasan) was purchased from TargetMol (Shanghai, China).

### 2.2 Jellyfish venom extraction and preparation

Live jellyfish specimens were identified as *Chrysaora chinensis* and were obtained from Beihai Zuohai Ocean Technology Co., Ltd. Jellyfish were collected from the coastal waters of Guangxi, China. The excised jellyfish tentacles were continuously stirred at 4 °C for 72 h to induce autolysis. The autolysate was centrifuged at 10,000 *g* for 15 min at 4 °C. The supernatant was dialyzed overnight against 1× PBS buffer, followed by another centrifugation at 10,000 *g* for 15 min at 4 °C. The resulting supernatant was collected to obtain the tentacle extract.

The total protein concentration of the jellyfish tentacle extract was determined using a BCA protein assay kit with bovine serum albumin as the standard. Absorbance was measured at 562 nm. The protein concentration of the prepared stock extract was calculated to be 2.50 mg/mL. All *in vivo* doses were normalized to total protein content and expressed as mg venom protein/kg body weight.

### 2.3 Animals

Male C57BL/6J mice aged 6–8 weeks and weighing 24–26 g (Chengdu Yaokang Biotechnology Co., Ltd., Chengdu, China; License No. SYCK [Sichuan] 2020-0034) were housed at the Hui Medicine Modernization Engineering Technology Research Center of Ningxia Medical University (Yinchuan, China). All animal experiments were approved by the Animal Ethics Committee of Ningxia Medical University and conducted in accordance with relevant institutional and national guidelines for the care and use of laboratory animals.

### 2.4 Establishment of the jellyfish envenomation model and experimental design

A jellyfish envenomation model was established in C57BL/6J mice via tail vein injection. After 1-week acclimatization, the animals were given two intravenous injections of jellyfish venom (11 mg/kg per injection) via the tail vein, with a 30-min interval between the injections. The 40 mice were equally randomized to five groups: (1) Control group (saline), (2) Venom group (venom + saline), (3) Edaravone (Eda) group (venom + Eda 10 mg/kg), (4) Low-dose APS group (venom + APS 500 mg/kg), and (5) High-dose APS group (venom + APS 1000 mg/kg). Drug administration was initiated 3 days prior to venom injection and continued thereafter. Eda was administered daily via intraperitoneal injection, and APS was administered daily via oral gavage. Drug administration was initiated on the day of venom injection and continued for 7 consecutive days.

To validate the target mechanism of APS, a separate mechanistic validation experiment was conducted using the Caspase-1 specific inhibitor VX-765. Following the same envenomation protocol, another cohort of 40 mice was randomly divided into five groups (n = 8/group): (1) Control group, (2) Venom group, (3) APS group (venom + APS 1000 mg/kg via oral gavage), (4) VX-765 group (venom + VX-765 50 mg/kg via intraperitoneal injection), and (5) Combination group (venom + APS 1000 mg/kg + VX-765 50 mg/kg). Drug administration commenced on the day of venom injection and continued for 7 consecutive days.

### 2.5 Preparation and characterization of crude astragalus polysaccharides

Radix Astragali was purchased from Ningxia Jintaiyang Co., Ltd. and authenticated according to the Chinese Pharmacopoeia 2020 edition. Astragalus crude polysaccharides were prepared by water extraction followed by ethanol precipitation. Briefly, dried Astragalus slices were pulverized and passed through a 40-mesh sieve. The powder, 300 g, was extracted with distilled water at a solid-to-liquid ratio of 1:10, w/v, under ultrasonication at 55 °C–65 °C for 2 h. The extraction was repeated three times. The combined aqueous extracts were concentrated under reduced pressure at 55 °C to approximately one-tenth of the original volume. After cooling to room temperature, absolute ethanol was slowly added with stirring to achieve a final ethanol concentration of 80%, v/v. The mixture was left standing at room temperature for 24 h, and the precipitate was collected by filtration and dried at 50 °C to constant weight to obtain Astragalus crude polysaccharides. Before administration, the crude polysaccharide preparation was dissolved in sterile normal saline, filtered through a 0.22 μm membrane, and freshly prepared. The same batch of crude APS was used throughout all pharmacodynamic experiments, and the administered dose was calculated based on the mass of the crude polysaccharide preparation.

The total polysaccharide content was determined using the phenol-sulfuric acid method with D-glucose as the reference standard. Briefly, glucose standard solutions ranging from 0 to 100 μg/mL or appropriately diluted APS sample solution were mixed with 5% phenol solution and concentrated sulfuric acid. After incubation at room temperature for 10 min and then at 40 °C for 15 min, the absorbance was measured at 485 nm. The total polysaccharide content was calculated as glucose equivalents based on the glucose standard curve.

### 2.6 Morris water maze test

Spatial learning and memory were assessed using a Morris water maze. The apparatus consisted of a circular black pool (diameter: 120 cm, height: 50 cm) filled with water (depth: 30 cm, temperature: 23 °C ± 1 °C). On day 1, the mice underwent a 5-min free swimming session without the platform to habituate to the environment. Place navigation training was carried out on day 2–4, during which an escape platform was submerged 1 cm below the water surface. Each mouse performed four trials per day (one starting from each quadrant), with a maximum trial duration of 60 s. On day 5, a probe trial was conducted after removing the platform, and the mice were released from the opposite quadrant of the original platform and allowed to swim freely for 45 s. Escape latency (time to reach the platform location, seconds) and path length (distance swum, meters) were recorded and analyzed using an automated tracking system.

### 2.7 Open field test

Anxiety-like behavior was assessed using an open field test. The apparatus was a square arena (50 cm × 50 cm × 40 cm) with the floor divided into 25 equal squares. The central zone was defined as the central 9 squares (30 cm × 30 cm). Testing was carried out in a quiet room. The mice were habituated to the testing room for 2 h prior to the experiment. Each mouse was placed in the center of the arena, and its behavior was recorded for 5 min using video tracking software. Parameters analyzed included total distance traveled (meters), average speed (m/s), and time spent in the central zone (seconds). The arena was thoroughly cleaned with 75% ethanol between trials to eliminate odor cues.

### 2.8 Brain tissue collection and processing

The mice were transcardially perfused with saline followed by 4% paraformaldehyde (PFA) in phosphate-buffered saline (PBS). The brain was removed, post-fixed in 4% PFA at 4 °C for 24 h, fixed in, and rinsed under running tap water for 12 h. The brain tissue was then dehydrated through a graded series of ethanol solutions (70%, 80%, 90%, 95% and 100%), cleared in xylene (two changes, 15 min each), infiltrated with molten paraffin wax (60 °C) for 45 min, and paraffin embedded. After solidification at room temperature, the brain tissue was sliced to 6–8 μm thick coronal sections using a microtome, mounted on glass slides, dried in a 60 °C oven for 2 h, and stored at room temperature for use.

### 2.9 Hematoxylin and eosin (H&E) staining

The paraffin-embedded brain sections were deparaffinized in xylene (two changes, 5 min each), rehydrated through a graded ethanol series (100%, 95%, 85% and 75%) to distilled water, stained with Mayer’s hematoxylin for 3–5 min, rinsed in tap water, differentiated in 1% acid alcohol (1% HCl in 70% ethanol) for a few seconds, blued in tap water, and counterstained with eosin solution for 30 s. After staining, the sections were dehydrated through graded ethanol (75%, 85%, 95% and 100%), cleared in xylene (two changes), and coverslipped using a neutral balsam mounting medium for microscopic observation and analysis.

### 2.10 Western blot analysis

The brain tissue was homogenized in ice-cold RIPA lysis buffer containing 1 mM PMSF and protease/phosphatase inhibitors. The homogenate was centrifuged at 12,000 × g at 4 °C for 15 min. The supernatant was collected, and the protein concentration was determined using BCA assay. The protein sample (30 µg per lane) was mixed with 4X Laemmli sample buffer at a 4:1 ratio, denatured by boiling at 95 °C for 10 min, and separated by SDS-PAGE on 10% polyacrylamide gels. Electrophoresis was performed initially at 80 V until the sample entered the resolving gel, then at 120 V. Proteins were electrophoresed at 200 mA for 90 min and transferred onto methanol-activated PVDF membrane. The membrane was blocked with 5% (w/v) non-fat dry milk in TBST (Tris-buffered saline containing 0.1% Tween-20) at room temperature for 2 h, and then incubated at 4 °C overnight with primary antibodies diluted at 1:1500 (β-tubulin at 1:5000) in blocking buffer or 5% BSA/TBST. After washing with TBST for 10 min x 3, the membrane was incubated with appropriate horseradish peroxidase (HRP)-conjugated goat anti-rabbit secondary antibodies (1:10,000) diluted in blocking buffer at room temperature for 2 h. Following further 10-min TBST wash × 3, protein bands were visualized using an enhanced chemiluminescence detection kit and imaged. Band intensities were quantified using ImageJ (National Institutes of Health, Bethesda, MD, United States).

### 2.11 Quantitative real-time polymerase chain reaction (RT-qPCR)

Total RNA was extracted from the brain tissue using TRIzol reagent according to the manufacturer’s instructions. Briefly, the tissue was homogenized in TRIzol (4 °C, 60 Hz, 60 s). Chloroform was added for phase separation, and RNA was precipitated from the aqueous phase using isopropanol. The RNA pellet was washed with 75% ethanol, air-dried, and dissolved in RNase-free water. The RNA concentration and purity (A260/A280 ratio) were determined spectrophotometrically. First-strand cDNA was synthesized from 1 µg total RNA using a reverse transcription kit with oligo (dT) primers under the following conditions: 72 °C for 5 min, 42 °C for 60 min, and 72 °C for 10 min. RT-qPCR was performed using a SYBR Green master mix on a real-time PCR system. The thermal cycling conditions were: 95 °C for 15 min (initial denaturation/activation), followed by 40 cycles of 95 °C for 10 s (denaturation), 59 °C for 20 s (annealing), and 72 °C for 30 s (extension). Melting curve analysis was performed to confirm amplification specificity. Relative mRNA expression levels were calculated using the 2^−ΔΔCt^ method, with glyceraldehyde-3-phosphate dehydrogenase (GAPDH) mRNA as the endogenous reference control.

### 2.12 Enzyme-linked immunosorbent assay (ELISA)

Blood samples were collected from the orbital venous plexus of the mice at the time of sacrifice. The samples were allowed to clot at room temperature for 30 min and then centrifuged at 3,000 rpm for 15 min at 4 °C. The upper serum layer was collected and stored at −80 °C. The serum levels of IL-1β, IL-18, and TNF-α were quantified using specific mouse ELISA kits according to the manufacturer’s instructions. The optical density (OD) was measured at 450 nm using a microplate reader, and the concentrations of the inflammatory cytokines were calculated based on the standard curves.

### 2.13 Statistical analysis

Behavioral experiment data are expressed as mean ± standard error (x̄ ± SEM), and other experimental data are expressed as mean ± standard deviation (x̄ ± SD). Statistical analyses were performed using SPSS 26.0 (IBM Corp., Armonk, NY, United States). Intergroup differences were assessed by one-way analysis of variance (ANOVA) followed by the Least Significant Difference *post hoc* test for multiple comparisons. A *p*-value <0.05 was considered statistically significant. For the VX-765 mechanistic validation experiment, all five groups were first compared by one-way ANOVA followed by LSD *post hoc* tests. In addition, the four venom-challenged groups (Venom, APS, VX-765, and APS + VX-765) were analyzed by two-way factorial ANOVA with APS and VX-765 as fixed factors; the APS × VX-765 interaction and *post hoc* comparisons were used to assess whether co-administration produced additive effects beyond monotherapy.

## 3 Results

### 3.1 Preparation and basic characterization of crude astragalus polysaccharide

Crude astragalus polysaccharides were obtained by water extraction and ethanol precipitation. From 300.0000 g of dried Astragalus powder, 6.5430 g of crude polysaccharide preparation was obtained, corresponding to an extraction yield of 2.18%. The total polysaccharide content, determined by the phenol-sulfuric acid method and expressed as glucose equivalents, was 38.35%. The same batch of crude APS was used for all subsequent *in vivo* experiments.

### 3.2 APS attenuates jellyfish envenomation-induced anxiety and locomotor deficits in the open field test

**FIGURE 1:**
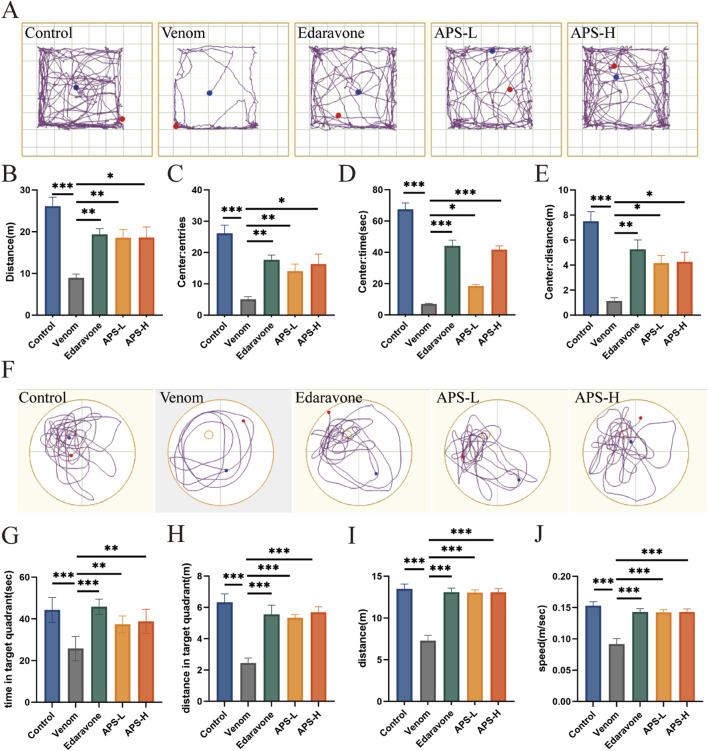
APS ameliorates jellyfish envenomation-induced anxiety-like behavior and cognitive deficits in mice. **(A)** Representative movement trajectories in open field test (OFT). **(B–E)** Quantitative analysis of OFT parameters: **(B)** Total distance traveled, **(C)** Number of center entries, **(D)** Time spent in center zone, **(E)** Distance traveled in center zone. Jellyfish envenomation (Venom group) significantly reduced all parameters compared to Control, indicating increased anxiety-like behavior and reduced exploration. Both Edaravone (Eda) and APS (both low and high doses) treatments significantly reversed these deficits. **(F)** Representative swimming paths during the Morris water maze (MWM) probe trial. **(G–J)** Quantitative analysis of MWM performance: **(G)** Time spent in target quadrant, **(H)** Distance traveled in target quadrant, **(I)** Total distance traveled, **(J)** Swimming speed. The Venom group exhibited significant impairment in spatial memory **(G,H)** and locomotor activity/exploration **(I,J)** compared to Control. Eda and APS treatments significantly restored spatial memory performance and locomotor activity. Data are mean ± SEM (n = 6). *P < 0.05, **P < 0.01, ***P < 0.001, vs. Venom group.

Representative movement trajectories revealed distinct behavioral patterns between the experimental groups (Figure 1A). The mice subjected to jellyfish envenomation (Venom group) exhibited significantly reduced total distance traveled compared to controls (*p* < 0.001; Figure 1B), indicating locomotor impairment. This deficit was significantly ameliorated by Eda (*p* < 0.01 vs. Venom) and both doses of APS (APS-L: *p* < 0.01; APS-H: *p* < 0.05 vs. Venom).

Concurrently, venom exposure induced marked anxiety-like behaviors, characterized by decreased center zone entries (*p* < 0.001 vs. Control; Figure 1C), reduced time in the center (*p* < 0.001 vs. Control; Figure 1D), and diminished center distance traveled (*p* < 0.001 vs. Control; Figure 1E). Both Eda (*p* < 0.01 vs. Venom) and APS treatments (APS-L: *p* < 0.05; APS-H: *p* < 0.05 vs. Venom) effectively reversed these anxiety-related parameters, with APS-H showing superior efficacy in restoring exploratory activity.

### 3.3 APS ameliorates venom-induced spatial memory impairment independent of locomotor dysfunction

Spatial memory was assessed in the Morris water maze probe trial. Representative swimming paths demonstrated that Control mice focused exploration on the target quadrant, while the Venom group displayed random, non-directed navigation (Figure 1F).

Quantitative analysis confirmed severe spatial memory deficits in Venom group, evidenced by significantly less time spent in the target quadrant (*p* < 0.001 vs. Control; Figure 1G) and reduced distance traveled within it (*p* < 0.001 vs. Control; Figure 1H). Eda (*p* < 0.001 vs. Venom) and both APS doses (*p* < 0.01 vs. Venom) significantly restored these memory indices.

To dissociate cognitive deficits from motor impairment, locomotor activity during the probe trial was analyzed. The Venom group showed reduced total distance traveled (*p* < 0.001 vs. Control; Figure 1I) and decreased swimming speed (*p* < 0.001 vs. Control; Figure 1J). Eda (*p* < 0.001 vs. Venom) and APS treatments (*p* < 0.001 vs. Venom) normalized both locomotor parameters, confirming that spatial memory improvement was independent of motor function.

### 3.4 Histopathological examination reveals hippocampal injury and adjacent hemorrhage

**FIGURE 2:**
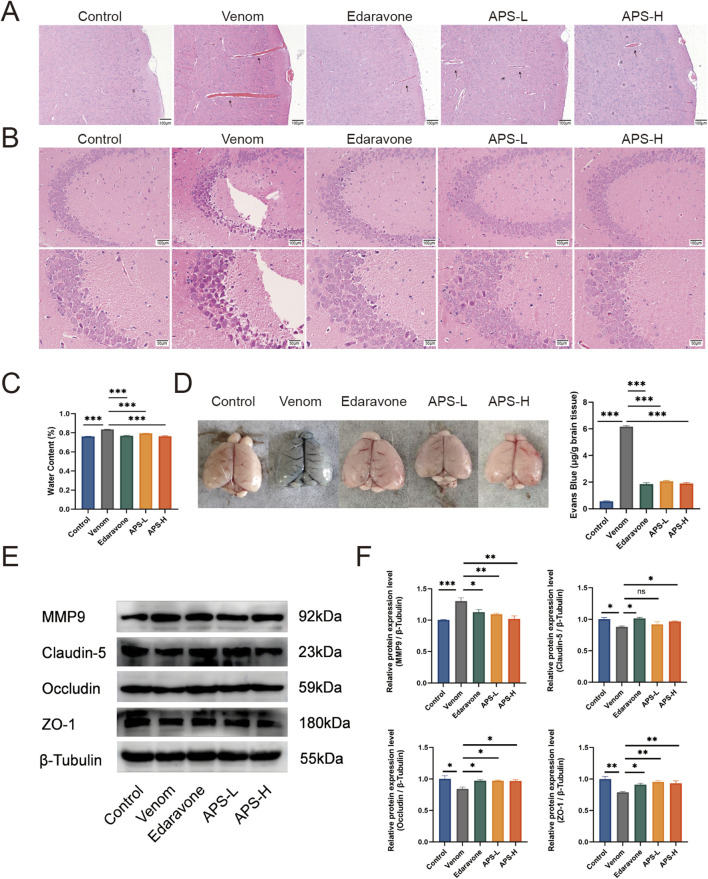
APS protects against hippocampal histopathological damage, hemorrhage, edema, and blood-brain barrier disruption induced by jellyfish venom. **(A)** Representative H&E-stained coronal brain sections (scale bar = 100 µm) highlighting the hippocampal CA3 region and adjacent areas. The neuronal architecture remained intact in Control mice, while severe pyramidal cell layer disorganization, neuronal loss, significant tissue edema, and prominent hemorrhagic foci in the tissue adjacent to the hippocampus were observed in Venom group (black arrowheads). Eda and APS treatments ameliorated these pathological changes. **(B)** Higher magnification (scale bar = 50 µm) of the CA3 region. **(C)** Quantification of brain water content (wet/dry weight ratio) confirming significant edema in the Venom group, attenuated by treatments. **(D)** Evans Blue (EB) extravasation assessment. Top: Representative images of EB-stained brains showing pronounced blue discoloration in Venom group. Bottom: Quantitative analysis of EB extravasation confirming severe BBB disruption in Venom group, which was significantly reduced by Eda and APS treatments. **(E)** Representative Western blots of TJ proteins (ZO-1, Occludin and Claudin-5), MMP9, and loading control (β-tublin). **(F)** Densitometric quantification. Venom challenge significantly downregulated TJ proteins and upregulated MMP9 vs. Control. Treatments significantly restored TJ protein expression and suppressed MMP9, with APS-H showing superior efficacy on Claudin-5 and MMP9. Data are mean ± SD (n = 3). *P < 0.05, **P < 0.01, ***P < 0.001 Venom (V) group. “ns” indicates no statistically significant difference compared with the Venom group.

To investigate the structural basis underlying the observed behavioral deficits, we examined hippocampal histopathology by H&E staining, focusing on the CA3 region, knowing that it is particularly vulnerable to neurotoxic insults (Figures 2A,B). Histological analysis revealed that Control mice displayed normal hippocampal cytoarchitecture with densely packed pyramidal neurons arranged in well-organized layers and intact cellular morphology, while the mice in Venom group exhibited severe pathological alterations within the hippocampus, including marked disruption of the pyramidal cell layer organization with extensive neuronal loss resulting in prominent areas of cellular depletion. Critically, elongated hemorrhagic foci were readily observable in the brain tissue adjacent to the hippocampal formation in sections from Venom-treated mice (Figure 2A), indicating significant vascular damage in regions proximate to the hippocampus. Additionally, significant tissue edema was evident within the hippocampus, characterized by widened intercellular spaces and increased neuropil vacuolization, suggesting compromised BBB integrity and neuroinflammation. This edema was quantitatively confirmed by a significant increase in brain water content (wet/dry weight ratio) in Venom group compared to all other groups (*p* < 0.001; Figure 2C). Remarkably, treatment with Eda substantially ameliorated these histopathological changes, preserving neuronal density, reducing edematous changes within the hippocampus, and mitigating adjacent hemorrhage. Similarly, significant neuroprotective effects were observed in both APS-L and APS-H groups, as represented by improved pyramidal cell layer organization, reduced neuronal loss, attenuated tissue edema within the hippocampus, and diminished hemorrhagic foci in adjacent regions compared to Venom group. The degree of histological improvement appeared to be dose-dependent, though this protective effect seemed more pronounced in APS-H group. These histopathological findings provide structural evidence supporting our behavioral observations and suggest that APS exerted its cognitive-enhancing and anxiolytic effects, at least in part, through preservation of hippocampal neuronal integrity, mitigation of venom-induced neuroinflammatory processes, and protection of the surrounding neurovasculatures.

### 3.5 APS attenuates venom-induced BBB disruption via preservation of TJ proteins and reduction of MMP9

The observed histopathological evidence of edema within the hippocampus and hemorrhage in adjacent regions strongly indicated widespread BBB compromise. To directly evaluate BBB integrity and the molecular mechanisms underlying venom-induced disruption, we employed Evans blue (EB) extravasation and assessed key TJ proteins and MMP9. Gross examination of EB-stained brains revealed pronounced blue discoloration in Venom group, indicative of substantial albumin-bound EB leakage into the brain parenchyma, which was significantly attenuated in all treatment groups (Figure 2D). Quantitative analysis demonstrated a highly significant increase in EB extravasation in Venom group compared to Control, Eda, APS-L, and APS-H groups (all *p* < 0.001; Figure 2D), corroborating the severe and widespread BBB dysfunction suggested by histology (edema and hemorrhage) and brain water content measurements.

Quantitative immunoblotting revealed that venom intoxication significantly compromised BBB integrity, as evidenced by coordinated downregulation of TJ proteins and concomitant MMP9 upregulation in the hippocampal tissue. In venom-challenged (V) mice, Occludin was reduced significantly (vs. control, *p* < 0.001), claudin-5 was reduced significantly (vs. control, *p* < 0.01), and ZO-1 was lost markedly (vs. control, *p* < 0.001), which were paralleled by MMP9 elevation (*p* < 0.001). Therapeutic interventions dose-dependently reversed these perturbations: Eda and APS treatments restored Occludin to near-baseline levels (Eda: *p* < 0.01, APS-L: *p* < 0.01, APS-H: *p* < 0.01 vs. V), while APS-H demonstrated superior efficacy in normalizing claudin-5 (*p* < 0.05 vs. V) and suppressing MMP9 (*p* < 0.01 vs. V) – outperforming both APS-Land Eda. Critically, this molecular reconstitution of TJ complexes strongly correlated with attenuation of the histopathological damage, establishing MMP9 inhibition and TJ protein stabilization as the mechanistic basis for APS-mediated BBB protection.

### 3.6 PCR analysis reveals APS-mediated transcriptional suppression of inflammasome components

**FIGURE 3:**
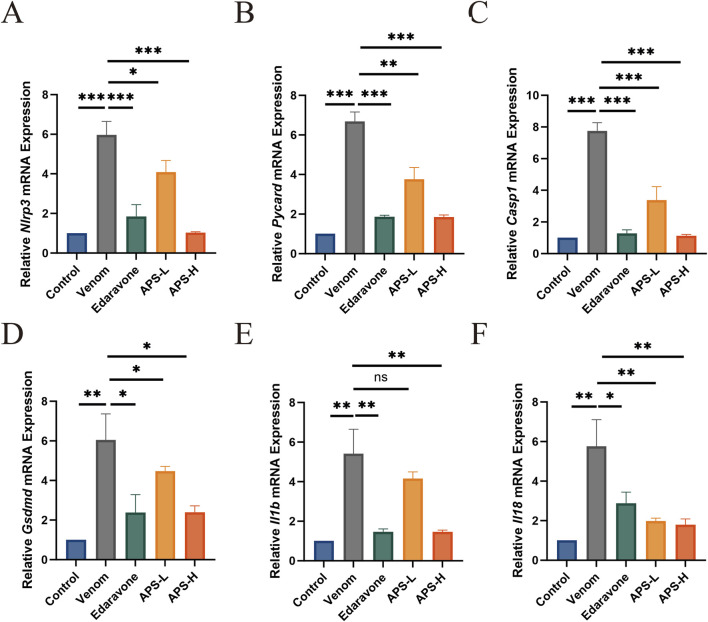
APS suppresses venom-induced inflammasome activation at the transcriptional level. RT-qPCR analysis of hippocampal mRNA expression: **(A)** *Nlrp3*, **(B)** *Pycard*, **(C)** *Casp1*, **(D)** *Gsdmd*, **(E)** *Il1b*, **(F)** *Il18*. Jellyfish envenomation (Venom group) significantly upregulated all transcripts compared to Control. Eda and APS treatments (APS-L and APS-H) significantly attenuated these increases, with APS-H showing the most potent effects. Data are mean ± SD (n = 6). *P < 0.05, **P < 0.01, ***P < 0.001 vs. Venom (V) group. “ns” indicates no statistically significant difference compared with the Venom group.

Complementary to our protein-level analyses, RT-qPCR assessment of the hippocampal tissue revealed significant transcriptional dysregulation in venom-challenged mice compared to Control group (Figure 3). We observed marked upregulation in the mRNA expression of key inflammasome components and pro-inflammatory cytokines: *Nlrp3* (*p* < 0.001), *Pycard* (encoding ASC; *p* < 0.001), Caspase-1 (*p* < 0.001), *Gsdmd* (*p* < 0.01), *Il1b* (*p* < 0.01), and IL-18 (*p* < 0.01) (Figures 3A–F). This comprehensive transcriptional activation underscores the engagement of pathways driving neuroinflammation at the gene expression level.

Both Eda and APS interventions significantly attenuated these transcriptional responses. APS-H demonstrated superior efficacy, significantly normalizing the elevated mRNA levels of *Il1b* (*p* < 0.01 vs. Venom), *Il18* (*p* < 0.01), *Casp1* (*p* < 0.001), and *Gsdmd* (*p* < 0.05) (Figures 3C–F). APS-L produced significant but intermediate suppression of *Gsdmd* (*p* < 0.05) and *Pycard* (*p* < 0.001) mRNA expression (Figures 3B,D). Both APS doses also significantly reduced *Nlrp3* mRNA (*p* < 0.05 for APS-L, *p* < 0.001 for APS-H) (Figure 3A).

Cross-platform validation established coherent regulation across molecular tiers. Notably, *Il1b*, *Il18*, and *Gsdmd* showed parallel downregulation at both mRNA (Figures 3D–F) and protein levels. This congruence was exemplified by the venom-induced elevation in *Gsdmd* mRNA corresponding to increased GSDMD-N protein, both of which were significantly attenuated by APS-H treatment.

### 3.7 APS inhibits jellyfish venom-induced pyroptosis execution at the protein level

**FIGURE 4:**
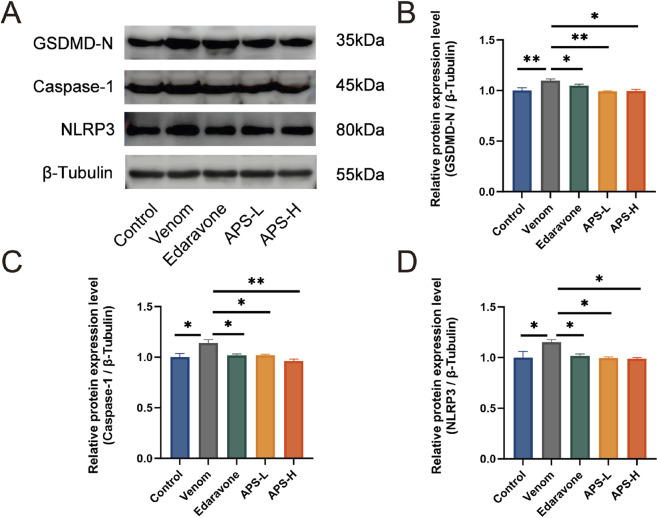
APS inhibits venom-induced pyroptosis execution at the protein level. **(A)** Representative Western blots for GSDMD-N, Caspase-1, NLRP3, and β-tubulin. **(B–D)** Densitometric quantification of GSDMD-N (B), Caspase-1 **(C)**, and NLRP3 **(D)** protein levels. Venom challenge significantly increased protein levels of NLRP3, Caspase-1, and GSDMD-N compared to Control. Eda and APS treatments significantly reversed these increases, confirming suppression of the canonical pyroptotic cascade. Data are mean ± SD (n = 3). *P < 0.05, **P < 0.01, ***P < 0.001 vs. Control group.

Quantitative analysis of the key pyroptosis markers (Figures 4A–D) revealed significant alterations in response to experimental treatments. Caspase-1 activation was markedly elevated in the venom group, exhibiting a significant increase compared with the Control group (*p* < 0.01). All intervention groups (Eda, APS-L and APS- H) demonstrated dose-dependent suppression of Caspase-1 cleavage, with APS-H treatment showing the most pronounced reduction (*p* < 0.001). Similarly, the mature form of GSDMD-N was significantly upregulated in V group (*p* < 0.001), while interventions effectively attenuated this effect. Notably, APS-H group reduced GSDMD-N levels (*p* < 0.001), indicating inhibition of pore-forming executioner protein formation. NLRP3 inflammasome assembly followed an identical trend: M group displayed robust NLRP3 accumulation (*p* < 0.01), whereas all therapeutic regimens normalized its expression to near-baseline levels (vs. M, p < 0.01). Critically, the coordinated downregulation of NLRP3, Caspase-1, and GSDMD-N across the three treatment groups (*p* < 0.05) confirmed the targeted suppression of the canonical pyroptotic cascade.

### 3.8 APS significantly attenuate venom-induced neuroinflammation

**FIGURE 5:**
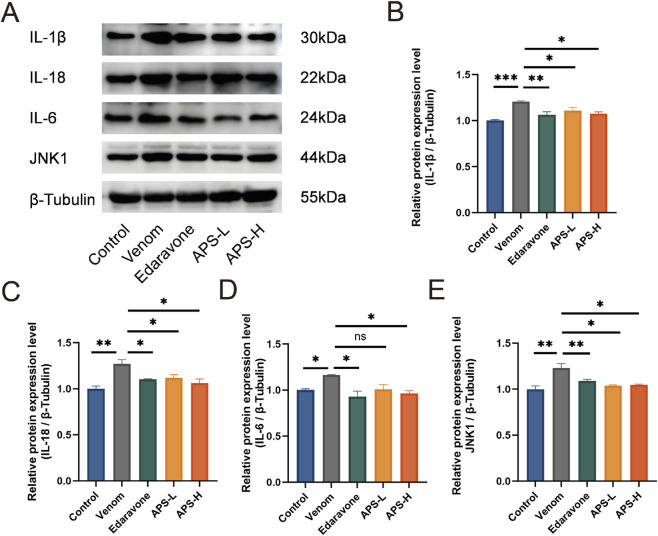
APS attenuates venom-induced neuroinflammatory protein expression. **(A)** Representative Western blots for IL-1β, IL-18, IL-6, JNK1, and β-tubulin. **(B–E)** Densitometric quantification of IL-1β **(B)**, IL-18 **(C)**, IL-6 **(D)**, and JNK1 **(E)** protein levels. Venom challenge significantly increased levels of pro-inflammatory mediators. APS treatments, particularly APS-H, significantly suppressed these increases and demonstrated superior efficacy to Eda in reducing JNK1 and IL-6. Data are mean ± SD (n = 3). *P < 0.05, **P < 0.01, ***P < 0.001 vs. Control group. “ns” indicates no statistically significant difference compared with the Venom group.

Hippocampal analysis (Figures 5A–E) revealed pronounced upregulation of the key pro-inflammatory mediators in envenomated mice, with IL-1β expression increasing (*p* < 0.001) and IL-18 rising (*p* < 0.001) vs. Control. APS intervention dose-dependently suppressed these perturbations, where APS-H normalized IL-1β to near-basal levels (*p* < 0.05 vs. venom group) and reduced IL-18 overexpression (*p* < 0.01). Critically, JNK1 activation, a central hub in MAPK-mediated neuroinflammation, was markedly amplified post-envenom ation (*p* < 0.001), an effect most potently reversed by APS-H (*p* < 0.01). IL-6 exhibited isoform-specific dysregulation, with the variant showing maximal venom-induced elevation (*p* < 0.05) which was effectively mitigated by APS-H.

### 3.9 VX-765 validates that APS exerts neuroprotection primarily by targeting the caspase-1-dependent pyroptosis pathway

To establish a causal relationship between Caspase-1-mediated pyroptosis and the neuroprotective effects of APS, we employed the Caspase-1-specific inhibitor VX-765 as a mechanistic probe.

**FIGURE 6:**
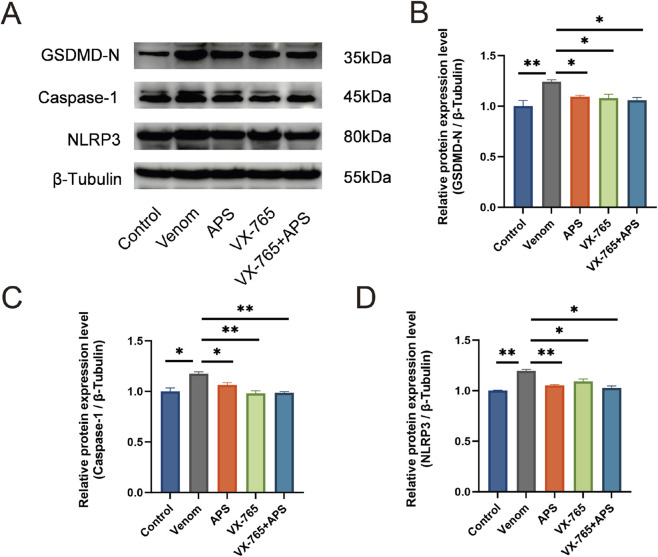
VX-765 confirms the dependence of APS neuroprotection on the Caspase-1 pyroptosis pathway at the protein level. **(A)** Representative Western blots for NLRP3, Cleaved Caspase-1, GSDMD-N, and β-Tubulin in brain tissue. **(B–D)** Densitometric quantification of NLRP3 **(B)**, Cleaved Caspase-1 **(C)**, and GSDMD-N **(D)**. Data are mean ± SD (n = 6). *P < 0.05, **P < 0.01. ns, no significant difference.

Western blot analysis of brain tissue demonstrated that jellyfish envenomation markedly upregulated NLRP3 expression, Caspase-1 cleavage, and GSDMD-N formation compared with the Control group (*p* < 0.01; Figures 6A–D). Treatment with APS, VX-765, or their combination each significantly suppressed all three pyroptotic markers relative to the Venom group (*p* < 0.05). Critically, the inhibitory efficacy of APS was equivalent to that of the Caspase-1-specific inhibitor VX-765 (*p* > 0.05), and combining the two agents produced no additive suppression beyond APS monotherapy (*p* > 0.05).

**FIGURE 7:**
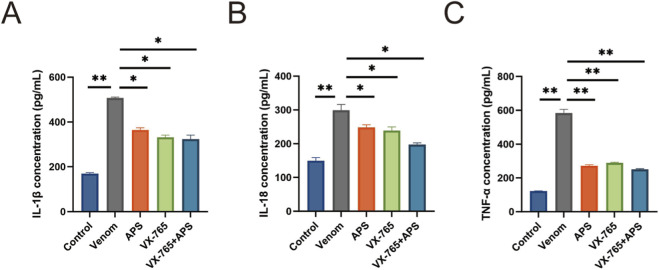
VX-765 confirms the dependence of APS neuroprotection on the Caspase-1 pyroptosis pathway at the cytokine level. **(A–C)** ELISA quantification of serum IL-18 **(A)**, IL-1β **(B)**, and TNF-α **(C)**. Data are mean ± SD (n = 6). *P < 0.05, **P < 0.01. ns, no significant difference.

ELISA analysis of peripheral serum revealed consistent results at the cytokine level (Figures 7A–C). Venom challenge significantly elevated IL-1β, IL-18, and TNF-α compared with the Control group (*p* < 0.01). All three treatment regimens effectively reduced circulating levels of these cytokines (*p* < 0.05 vs. Venom). Likewise, no statistically significant differences were observed between APS and VX-765 monotherapies (*p* > 0.05), nor between the combination group and APS alone (*p* > 0.05).

The equivalent efficacy of APS and the Caspase-1-specific inhibitor VX-765, together with the absence of synergy upon their combination, collectively demonstrates that the neuroprotective and anti-inflammatory effects of APS are primarily mediated through inhibition of the canonical Caspase-1-dependent pyroptosis pathway.

## 4 Discussion

Neurotoxicity induced by jellyfish stings is a rapidly progressing marine health threat whose mechanisms have not been fully elucidated (Carneiro et al., 2011; Prakash et al., 2020). In the present study, we used an integrated *in vivo* experimental strategy combining behavioral assessment, histopathological evaluation, BBB permeability analysis, and molecular validation of inflammatory pyroptosis. The major findings are as follows: (1) jellyfish venom exposure induced marked cognitive and behavioral abnormalities, accompanied by hippocampal injury, cerebral edema, hemorrhage, and BBB disruption; (2) MMP9 upregulation and tight junction protein loss were observed after venom exposure, suggesting that MMP9-associated BBB impairment may be an important pathological event in jellyfish venom-induced neurotoxicity; (3) to our knowledge, this is the first study demonstrating that crude astragalus polysaccharides (APS) effectively ameliorate jellyfish venom-induced cognitive dysfunction and nervous system injury; and (4) APS-mediated neuroprotection was closely associated with inhibition of the NLRP3/Caspase-1/GSDMD pyroptotic pathway, which was further supported by pharmacological validation using the Caspase-1-specific inhibitor VX-765. These findings provide new experimental evidence for understanding the neurotoxic mechanisms of jellyfish envenomation and suggest that preserving BBB integrity and suppressing Caspase-1/GSDMD-mediated pyroptosis may represent promising therapeutic strategies for marine envenomation-associated neurological injury.

Because TCMSP-based network pharmacology mainly reflects low-molecular-weight constituents of Astragalus membranaceus rather than the macromolecular structural features of Astragalus crude polysaccharides, these computational results cannot be interpreted as direct APS-target evidence. Accordingly, the network pharmacology findings are provided only in Supplementary Table S1 for exploratory pathway prioritization, whereas the mechanistic conclusions of this study are based on the *in vivo* BBB, inflammatory pyroptosis, and VX-765 validation experiments.

Current research on jellyfish venom neurotoxicity has mainly focused on direct cellular damage (Chiang and Castillo, 2014), ion channel regulation (Choudhary et al., 2021), and mitochondrial injury (Mirshamsi et al., 2017), while BBB damage has not been sufficiently studied. This study first verified the neurotoxicity of jellyfish venom through behavioral tests. The open field test showed increased anxiety-like behavior in venom group mice, while the Morris water maze test reflected impaired spatial memory and decreased exploratory initiative, suggesting that jellyfish venom caused significant cognitive dysfunction and anxiety. After Astragalus polysaccharide treatment, the aforementioned cognitive impairment and behavioral abnormalities showed a certain degree of improvement.

Wet-dry brain weight experiments and H.E. staining results showed brain edema, disordered neuronal arrangement, and obvious hemorrhage in the venom group, suggesting that jellyfish venom induced cerebral edema and hemorrhage. Evans Blue staining images and quantitative analysis visually confirmed increased blood-brain barrier permeability. The expression of tight junction proteins ZO-1, Occludin, and Claudin-5 was downregulated, further confirming that BBB barrier function was disrupted and its permeability significantly increased. Astragalus polysaccharide treatment significantly improved hippocampal tissue damage, reduced cerebral edema, and partially restored tight junction protein expression.

How exactly is the BBB disrupted? What is its molecular mechanism? This study found that MMP9 protein expression in the mouse hippocampus was significantly upregulated, suggesting its possible involvement in the BBB injury process. As a zinc-dependent endopeptidase, MMP9 can be activated under pathological stimulation and specifically degrade extracellular matrix and basement membrane components (Muneer et al., 2016). Its role in mediating BBB disruption (Turner and Sharp, 2016; Wu et al., 2022) and cognitive dysfunction (Jia et al., 2014; Miao et al., 2023) in various neurological diseases such as cerebral ischemia and traumatic brain injury has been widely confirmed. In this study, MMP9 upregulation occurred synchronously with tight junction protein loss, which is consistent with previous reports on MMP9 degradation of BBB structural proteins (Dhanda and Sandhir, 2018). Furthermore, MMP9 activation is often closely related to inflammatory responses (Li Y. et al., 2025). Inflammatory factors can induce MMP9 expression, while activated MMP9 promotes inflammation spread, thus forming a vicious cycle of increased inflammation and MMP9 activation. Crude astragalus polysaccharides can significantly inhibit MMP9 activation and restore tight junction protein expression, thereby reducing BBB disruption and cerebral edema, indicating that it exerts neuroprotective effects by regulating MMP9 downregulation and maintaining tight junction protein integrity to preserve BBB integrity, laying a solid foundation for subsequent inflammation mechanism research.

In various BBB disruption models, such as ischemic stroke, loss of barrier function has been widely confirmed to activate inflammatory cascade reactions within the neurovascular unit (Smith et al., 2022), and animal toxicology studies also indicate that disruption of neurovascular integrity can increase susceptibility to neuroinflammation (Kim et al., 2022). Based on the significant protective effect of crude astragalus polysaccharides on BBB integrity, we further investigated the inflammatory mechanisms involved in jellyfish venom-induced neural injury. Our results showed that jellyfish venom markedly upregulated the expression of the pro-inflammatory mediators IL-1β, IL-6, and IL-18, together with increased JNK1 expression, suggesting activation of stress-related MAPK inflammatory signaling and neuroinflammatory responses. JNK1, as an important member of the MAPK family, has been implicated in inflammatory signal transduction and inflammasome regulation, and its upregulation may contribute to the amplification of venom-induced neuroinflammation. Notably, crude astragalus polysaccharides significantly reduced JNK1 expression and suppressed the production of IL-1β, IL-6, and IL-18, indicating that its neuroprotective effect is closely associated with attenuation of MAPK-related inflammatory responses. These findings are consistent with previous reports showing that jellyfish venom can promote the release of inflammatory factors such as IL-6 (Wang et al., 2025), further supporting the notion that neuroinflammation is an important pathological component of jellyfish venom-induced nervous system injury.

To further clarify the mechanisms underlying the release of inflammatory mediators, particularly the Caspase-1-dependent maturation of IL-1β and IL-18, we focused on the canonical pyroptosis pathway. Our experiments showed that jellyfish venom significantly upregulated NLRP3 expression, Caspase-1 cleavage, and GSDMD-N formation in hippocampal tissue, suggesting activation of the NLRP3/Caspase-1/GSDMD signaling pathway. Pyroptosis is an inflammatory form of programmed cell death characterized by Caspase-1-mediated cleavage of GSDMD. The generated N-terminal fragment, GSDMD-N, forms pores in the plasma membrane, leading to cell swelling, membrane rupture, and the release of inflammatory mediators (Wang et al., 2024), thereby amplifying neuroinflammatory responses and exacerbating tissue damage (Ding et al., 2022). The increased formation of GSDMD-N observed in this study was accompanied by elevated IL-1β and IL-18 levels, which is consistent with activation of canonical inflammasome-dependent pyroptosis. Notably, treatment with crude astragalus polysaccharides significantly inhibited this pyroptotic process, as evidenced by reduced NLRP3 expression, decreased Caspase-1 activation, suppressed GSDMD-N generation, and lower IL-1β and IL-18 levels. In addition, APS reduced IL-6 expression, suggesting a broader attenuation of venom-induced neuroinflammatory responses. These *in vivo* findings indicate that crude astragalus polysaccharides exert neuroprotective effects, at least in part, by inhibiting the NLRP3/Caspase-1/GSDMD pyroptosis pathway and reducing associated neuroinflammation.

To definitively establish the causal relationship between Caspase-1-mediated pyroptosis and the neuroprotective effects of APS, we introduced the Caspase-1 specific inhibitor VX-765. Our results demonstrated that VX-765 and APS exhibited comparable inhibitory efficacies on both systemic inflammatory cytokine release (serum IL-1β, IL-18, and TNF-α) and central inflammasome activation (brain NLRP3, Caspase-1, and GSDMD-N). Most importantly, the co-administration of APS and VX-765 did not yield a synergistic or additive effect. In pharmacological terms, the lack of an additive effect when two agents are combined strongly indicates that they act upon the same signaling pathway or share a converging mechanistic target. This critical validation confirms that the suppression of Caspase-1-dependent pyroptosis is not merely an epiphenomenon, but rather a primary, indispensable mechanism driving the neuroprotective efficacy of APS against jellyfish venom.

After jellyfish venom is injected into the human body through nematocysts, it rapidly enters the blood circulation and is distributed to various organs throughout the body via blood flow, causing direct acute damage to peripheral organs such as skin (Li et al., 2024) and heart (Li B. et al., 2025). Clinically, we have collected a case report of a marine training personnel who developed consciousness disorder and cerebral edema after jellyfish sting and ultimately died, indicating that jellyfish venom can cause severe or even fatal damage to the nervous system. The jellyfish venom poisoning model established in this study 7 days after modeling represents an important part of the mechanism of nervous system damage after jellyfish stings, focusing on hippocampal pathological changes and cognitive impairment. This study reached the following conclusions: Jellyfish venom causes BBB disruption by activating MMP9 to degrade tight junction proteins, subsequently activating the NLRP3/Caspase-1/GSDMD pyroptosis pathway, releasing inflammatory factors IL-1β, IL-6, and IL-18, ultimately leading to hippocampal neuronal damage and cognitive dysfunction; Crude astragalus polysaccharides exert neuroprotective effects by maintaining BBB integrity and inhibiting pyroptosis. This study mainly focused on changes in endothelial cell barrier function, while the direct effects of jellyfish venom on specific neural cells such as astrocytes or microglia need to be further clarified, and the interactions among components of the neurovascular unit and their roles in BBB damage also need to be explored in depth. Future research plans to include astrocytes and microglia to comprehensively analyze the overall changes in the neurovascular unit and more comprehensively elucidate the neural injury mechanism of jellyfish venom. Given the high incidence of jellyfish sting events and the severity of neurological sequelae, developing intervention strategies based on BBB protection and anti-pyroptosis has important public health significance, providing more effective treatment options for jellyfish sting patients.

## 5 Conclusion

In conclusion, this study provides preclinical evidence in a mouse model that APS attenuate jellyfish venom-induced neurotoxicity, with protective effects associated with preservation of BBB integrity and suppression of neuroinflammatory pyroptosis. APS treatment alleviated venom-induced anxiety-like behaviors, locomotor deficits, and spatial memory impairment, accompanied by improved hippocampal cytoarchitecture and reduced cerebral edema and hemorrhage. APS mitigated BBB disruption by reducing MMP9 upregulation and partially restoring tight junction proteins, including Occludin, Claudin-5, and ZO-1. APS also decreased JNK1 expression and suppressed activation of the NLRP3/Caspase-1/GSDMD pyroptotic pathway. Pharmacological validation using the Caspase-1 inhibitor VX-765 further supported the involvement of Caspase-1-dependent pyroptosis in APS-associated neuroprotection. These findings suggest that APS may serve as a candidate adjunctive therapeutic strategy for marine envenomation-associated neurological injury and highlight BBB dysfunction and Caspase-1/GSDMD-mediated pyroptosis as potential intervention targets. Further studies are warranted to identify the active constituents of crude jellyfish venom and crude APS, clarify the upstream events linking venom exposure to MMP9 activation and inflammasome signaling, and evaluate structurally defined APS fractions for therapeutic window, efficacy, and safety in delayed-treatment paradigms and clinically relevant envenomation models.

## Data Availability

The datasets presented in this study can be found in online repositories. The names of the repository/repositories and accession number(s) can be found in the article/Supplementary Material.
