# Supplementary figures and images for: Crude Astragalus polysaccharides ameliorate cognitive impairment by preserving blood-brain barrier integrity and suppressing GSDMD-mediated pyroptosis in jellyfish-envenomed mice
Source: Front Pharmacol. 2026 Jun 8;17:1853198. doi: 10.3389/fphar.2026.1853198 (PMC13284131; doi:10.3389/fphar.2026.1853198)

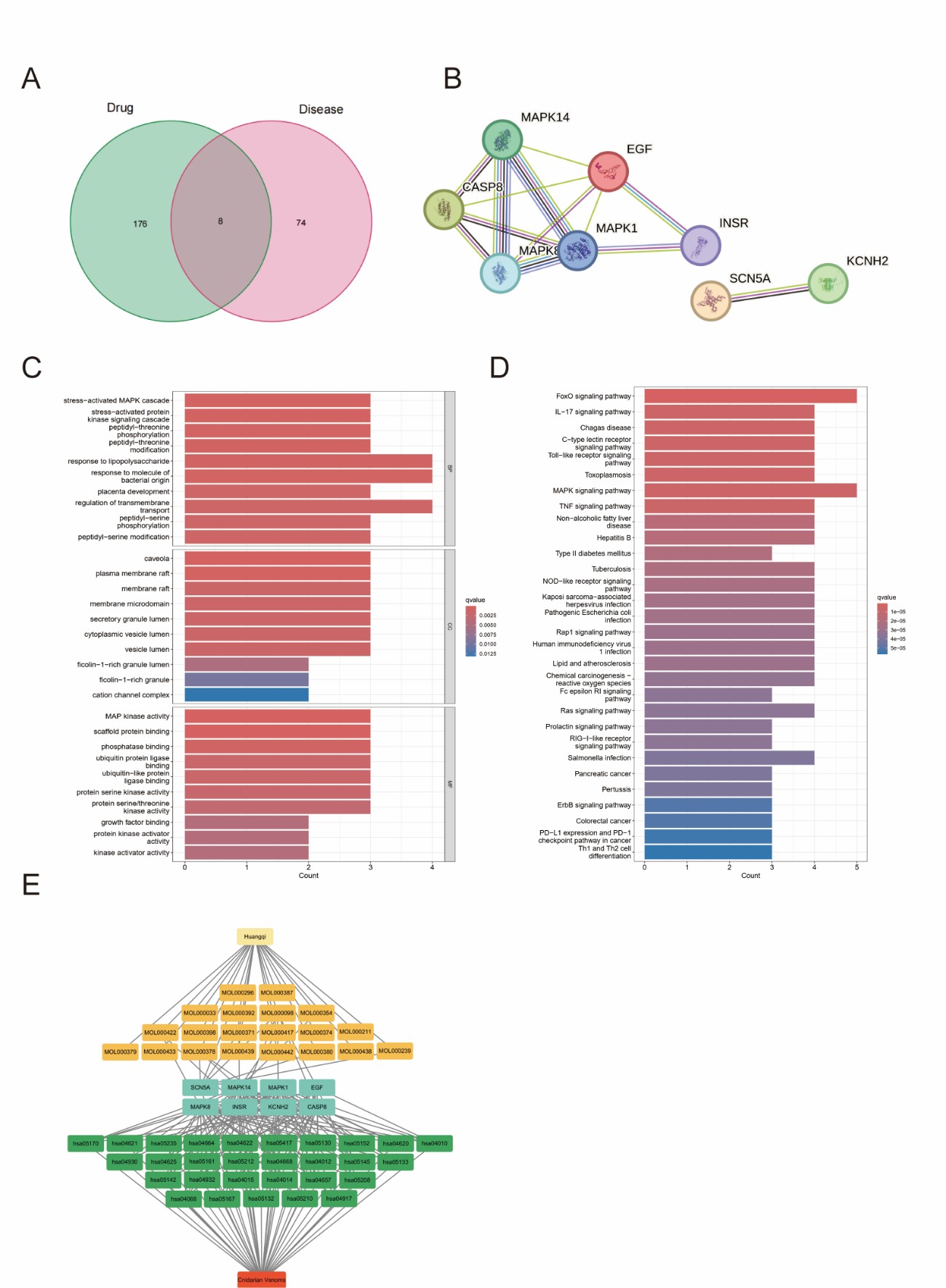

Supplement: Supplementary file 1 [file Image1.jpeg]
