# Supplementary material for: Crude Astragalus polysaccharides ameliorate cognitive impairment by preserving blood-brain barrier integrity and suppressing GSDMD-mediated pyroptosis in jellyfish-envenomed mice
Source: Front Pharmacol. 2026 Jun 8;17:1853198. doi: 10.3389/fphar.2026.1853198 (PMC13284131; doi:10.3389/fphar.2026.1853198)
Supplement: Supplementary file 2 [file DataSheet1.pdf]

S1 Exploratory network pharmacology analysis based on small-molecule constituents of Astragalus membranaceus

S1.1 Methods

S1.1.1 Screening of Astragalus membranaceus small-molecule constituents and target collection

The chemical constituents of *Astragalus membranaceus* were retrieved from the Traditional Chinese Medicine Systems Pharmacology Database and Analysis Platform (TCMSP). Compounds were screened using oral bioavailability (OB) ≥ 30% and drug-likeness (DL) ≥ 0.18 as selection criteria. The predicted or annotated protein targets of the eligible constituents were collected from TCMSP and standardized using the UniProt database. Duplicate targets were removed before subsequent analysis. These targets were defined as *A. membranaceus* small-molecule-associated targets, rather than APS-specific targets.

S1.1.2 Collection of jellyfish envenomation-related targets

Jellyfish envenomation-related targets were collected from GeneCards, OMIM, and DisGeNET databases using the keywords "jellyfish envenomation", "jellyfish venom", and "marine envenomation". After removing duplicate targets, the remaining genes were used as the jellyfish envenomation-related disease target set.

S1.1.3 Identification of overlapping targets and construction of the protein-protein interaction network

The overlapping targets between the A. membranaceus small-molecule-associated target set and the jellyfish envenomation-related target set were identified using Venn diagram analysis. These overlapping targets were used as candidate targets for exploratory pathway analysis. The overlapping targets were then submitted to the STRING database to construct a protein-protein interaction (PPI) network. The organism was set as "Homo sapiens", and the minimum required interaction score was set to 0.700. The PPI network was imported into Cytoscape 3.9.1 for visualization and topological analysis. Degree and betweenness centrality were used to evaluate the relative importance of network nodes.

S1.1.4 GO and KEGG enrichment analyses

Gene Ontology (GO) enrichment analysis and Kyoto Encyclopedia of Genes and Genomes (KEGG) pathway enrichment analysis were performed using the R package clusterProfiler under the "Homo sapiens" background. An adjusted P value < 0.05 was considered statistically significant. GO enrichment analysis included biological process, cellular component, and molecular function categories. KEGG enrichment analysis was used to identify candidate signaling pathways potentially associated with the overlapping targets. The enrichment results were visualized using bubble plots.

S1.1.5 Construction of the compound-target-pathway-disease network

The eligible A. membranaceus small-molecule constituents, overlapping targets, significantly enriched KEGG pathways, and the jellyfish envenomation disease node were integrated to construct a compound-target-pathway-disease network using Cytoscape 3.9.1. In the network,

node size and color intensity were used to reflect the topological importance of each node.

S1.2 Supplementary Results

A total of 17 eligible low-molecular-weight constituents of Astragalus membranaceus were retrieved from TCMSP according to the screening criteria of oral bioavailability (OB) ≥ 30% and drug-likeness (DL) ≥ 0.18. These constituents were associated with 176 predicted or annotated targets, while 74 jellyfish envenomation-related targets were collected from GeneCards, OMIM, and DisGeNET. Venn diagram analysis identified eight overlapping targets, including MAPK14, MAPK8, MAPK1, CASP8, INSR, EGF, SCN5A, and KCNH2 (Supplementary Figure S1A). PPI network analysis showed that MAPK-related nodes were centrally located within the network, with MAPK1, EGF, and MAPK14 showing relatively high topological relevance, while MAPK8/JNK1 linked the network to MAPK-associated inflammatory signaling (Supplementary Figure S1B). GO enrichment analysis indicated that the overlapping targets were mainly enriched in stress- and inflammation-related biological processes, including stress-activated MAPK cascade (GO:0051403, $P = 1.14 \times 10^{-6}$), response to lipopolysaccharide (GO:0032496, $P = 7.84 \times 10^{-6}$), and regulation of transmembrane transport (GO:0034762, $P = 2.76 \times 10^{-5}$), as well as membrane-associated cellular components such as caveolae (GO:0005901, $P = 3.69 \times 10^{-6}$) and membrane rafts (GO:0044853, $P = 1.05 \times 10^{-5}$) (Supplementary Figure S1C). KEGG enrichment analysis suggested that MAPK signaling pathway (hsa04010, $P = 8.56 \times 10^{-7}$), IL-17 signaling pathway (hsa04657, $P = 1.86 \times 10^{-5}$), and TNF signaling pathway (hsa04668, $P = 1.99 \times 10^{-5}$) were enriched among the overlapping targets (Supplementary Figure S1D). The compound-target-pathway-disease network further showed that several A. membranaceus small-molecule constituents were connected with MAPK-related targets, including MAPK14, MAPK8, and MAPK1, and inflammation-associated pathways (Supplementary Figure S1E). Collectively, this exploratory analysis suggested MAPK-related inflammatory signaling as a candidate pathway for subsequent in vivo validation. However, because this analysis was based on small-molecule constituents of A. membranaceus rather than Astragalus crude polysaccharides themselves, these results should not be interpreted as direct evidence that APS binds to or directly regulates these targets.

Supplementary Figure S1. Exploratory network pharmacology analysis based on small-molecule constituents of Astragalus membranaceus. (A) Venn diagram showing eight overlapping targets between jellyfish envenomation-related targets and predicted targets associated with A. membranaceus small-molecule constituents. (B) PPI network of the overlapping targets. (C) GO enrichment analysis of the overlapping targets. (D) KEGG pathway enrichment analysis showing enrichment of MAPK and inflammation-related signaling pathways. (E) Compound-target-pathway-disease network integrating.

S2. Protein quantification of jellyfish tentacle extract

The total protein concentration of the prepared Chrysaora chinensis tentacle extract was determined by BCA assay using bovine serum albumin as the standard. The stock protein

concentration was 2.50 mg/mL. All in vivo doses were normalized to total protein content and expressed as mg venom protein/kg body weight.
